# Therapeutic Potential of Emerging NAD+-Increasing Strategies for Cardiovascular Diseases

**DOI:** 10.3390/antiox10121939

**Published:** 2021-12-03

**Authors:** Noemi Rotllan, Mercedes Camacho, Mireia Tondo, Elena M. G. Diarte-Añazco, Marina Canyelles, Karen Alejandra Méndez-Lara, Sonia Benitez, Núria Alonso, Didac Mauricio, Joan Carles Escolà-Gil, Francisco Blanco-Vaca, Josep Julve

**Affiliations:** 1 Institut de Recerca i d’Investigació Biomèdica de l’Hospital de la Santa Creu i Sant Pau, IIB-Sant Pau, 08041 Barcelona, Spain; nrotllanv@santpau.cat (N.R.); mcamacho@santpau.cat (M.C.); ediarte@santpau.cat (E.M.G.D.-A.); mcanyelles@santpau.cat (M.C.); kmendez@santpau.cat (K.A.M.-L.); sbenitez@santpau.cat (S.B.); 2 Department of Biochemistry and Molecular Biology, Universitat Autònoma de Barcelona, 08193 Barcelona, Spain; mtondo@santpau.cat; 3 CIBER de Diabetes y Enfermedades Metabólicas Asociadas, CIBERDEM, 28029 Madrid, Spain; nalonso.germanstrias@gencat.cat (N.A.); dmauricio@santpau.cat (D.M.); 4 CIBER de Enfermedades Cardiovasculares, CIBERCV, 28029 Madrid, Spain; 5 Department of Biochemistry, Hospital de la Santa Creu i Sant Pau, IIB-Sant Pau, 08041 Barcelona, Spain; 6 Department of Endocrinology & Nutrition, Hospital Universitari Germans Trias i Pujol, 08916 Barcelona, Spain; 7 Department of Endocrinology & Nutrition, Hospital de la Santa Creu i Sant Pau, IIB-Sant Pau, 08041 Barcelona, Spain

**Keywords:** macrophage, aneurysm, atherosclerosis, ischemia/reperfusion, cardiomyopathy, heart failure, chemotherapy, clinical trials, COVID-19, niacinamide, vitamin B3, niacin, niagen, niaspan, mitochondria, tryptophan, diabetes, myocarditis, animal models, clinical trials

## Abstract

Cardiovascular diseases are the leading cause of death worldwide. Aging and/or metabolic stress directly impact the cardiovascular system. Over the last few years, the contributions of altered nicotinamide adenine dinucleotide (NAD+) metabolism to aging and other pathological conditions closely related to cardiovascular diseases have been intensively investigated. NAD+ bioavailability decreases with age and cardiometabolic conditions in several mammalian tissues. Compelling data suggest that declining tissue NAD+ is commonly related to mitochondrial dysfunction and might be considered as a therapeutic target. Thus, NAD+ replenishment by either genetic or natural dietary NAD+-increasing strategies has been recently demonstrated to be effective for improving the pathophysiology of cardiac and vascular health in different experimental models, as well as human health, to a lesser extent. Here, we review and discuss recent experimental evidence illustrating that increasing NAD+ bioavailability, particularly by the use of natural NAD+ precursors, may offer hope for new therapeutic strategies to prevent and treat cardiovascular diseases.

## 1. Introduction

Cardiovascular diseases are the leading cause of death worldwide according to the World Health Organization. Aging is a primary risk factor for many cardiovascular diseases. With the progressively aging population, the elevated prevalence of cardiovascular diseases undermines human health and longevity [1].

Mitochondrial dysfunction is a hallmark of metabolic decline during aging [2]. Nicotinamide adenine dinucleotide (NAD+) bioavailability decreases with age in a number of mammalian tissues, and evidence suggests that failing mitochondrial function is related to this decline [3,4]. Beyond aging, altered NAD+ metabolism levels have also been reported in obesity, diabetes mellitus, fatty liver disease, and hypertension [5]. NAD+ depletion is observed in several cardiovascular pathologies, including atherosclerosis, ischemia-reperfusion injury, cardiomyopathy, and heart failure (HF), which has prompted studies exploring its pathophysiological role in cardiovascular diseases [6]. In this regard, NAD+ decline prevention may represent an innovative therapeutic target in the management of cardiovascular diseases [7]. Accordingly, many studies have revealed that dietary administration of NAD+ precursors, such as tryptophan, nicotinic acid (NA, also termed niacin), nicotinamide (NAM), and nicotinamide riboside (NR), and its phosphorylated form, nicotinamide mononucleotide (NMN), efficiently raises the tissue NAD+ content in rodents and humans [8,9]. In particular, NAM, NMN, and NR administration has been reported to prevent obesity and improve glucose tolerance in mice [10,11,12,13,14] and favorably influenced fatty liver by reducing oxidative stress and inflammation in aged, diet-induced obese mice [15]. Conceivably, NAD+ precursors may protect the cardiovascular system [6,16].

NA is a vitamin B3 form that has been widely used in clinical practice to reduce the atherothrombotic cardiovascular risk by decreasing plasma triglycerides and increasing plasma high-density lipoprotein (HDL) cholesterol (HDL-C) [17]. NA interacts with the GPR109A receptor in immune cells, thereby blunting immune activation and protecting against inflammatory cell infiltration in age-related diseases [18]. Recent studies suggest that many physiological effects of NA on cardiovascular protection can be at least partly explained on the basis of its role as an NAD+ precursor [19]. However, a major problem with NA administration is adverse effects, which range from cutaneous flushing to effects linked to muscle, liver, and gastrointestinal toxicity [20]. Moreover, early reports supporting secondary cardiovascular preventive effects of NA were not confirmed by more recent, large prospective trials including 29,087 patients, the AIM-HIGH [21] and the HPS2-THRIVE [22], which were prematurely stopped due to a lack of efficacy in patients that were taking statins in the context of secondary prevention. Thus, these results do not, therefore, necessarily apply to primary cardiovascular prevention, but not such type of novel NA clinical trial seems to be planned at this point. In a recent study [23], NA also ameliorated wound healing and cardiac function after myocardial infarction via DP1-mediated M2 macrophage polarization and timely resolution of inflammation, thus suggesting that DP1 inhibition, as the agent used together with NA in the abovementioned large clinical trials, may attenuate the cardiovascular benefits of NA.

The potential benefits of other forms of vitamin B3 have also been studied, albeit more experimentally than at the clinical level. NAM, an amide vitamin B3 form, does not interact with the GPR109A receptor and is apparently free of NA-induced adverse side effects [7,8]. Treatment with both NAM and NA improved aneurysms in an experimental mouse model through GPR109A receptor-independent activity against this disease [24]. Unlike NA, NAM does not show strong lipid-lowering effects [19] but exerts antioxidant and anti-inflammatory effects [15,25]. Interestingly, NAM administration protected against beta pancreatic cell damage and loss and, thus, delayed the onset of diabetes mellitus type 1 in experimental models [26]. Although this effect was not reproduced in the ENDIT clinical trial [27], NAM administration at a dose of 1.2 g/m^2^ did not cause adverse effects (including flushing) similar to those attributed to NA. Recent data from our own group show that NAM prevents inflammation and atherosclerosis in experimental animals [25]. Similarly, some other related compounds, such as NR [28], NMN [9], and N-methylated NAM (me-NAM) [29,30], seem to protect against vascular damage.

Some evidence indicates that NAM or other related compounds that act as a source of NAD+ might be of interest in the context of cardiomyopathy and heart failure (HF) [31].

Thus, this comprehensive review provides updated, critically revised information on the potential therapeutic effects of boosting NAD+ metabolism in animal models of cardiovascular diseases and its potential translation into clinical practice. In particular, we mainly focus on the role of NAD+-increasing strategies based on the use of NAD+ precursors. Other determinants of NAD+ metabolism also lead to NAD+ elevations; however, owing to space limitations, genetically modified mouse models are not extensively discussed in this review. Furthermore, although various pharmacological strategies have also been reported to boost NAD+ levels, including the use of activators and inhibitors of NAD+ biosynthesis, we focus on the therapeutic potential of direct natural NAD+ precursors in cardiovascular health.

## 2. Preferential Physiological Sources of NAD+

NAD+ is an essential cofactor in all living cells [32]. Abundant amounts of this metabolite are found in different cellular compartments, including mitochondria, cytoplasm, and nuclei. NAD+ plays a key physiological role in modulating many biological processes and cellular metabolism pathways, including glycolysis, fatty acid β-oxidation, and the tricarboxylic acid cycle [33,34]. NAD+ exists in two forms—the oxidized form (NAD+) and the reduced form (NADH). The latter is generated by NAD+ accepting high-energy electrons from glycolytic and tricarboxylic acid intermediates and acts as a primary electron donor in ATP production by feeding electrons into complex I of the electron transport chain to drive mitochondrial oxidative phosphorylation [35].

### 2.1. NAD+ Sources

**Figure 1:**
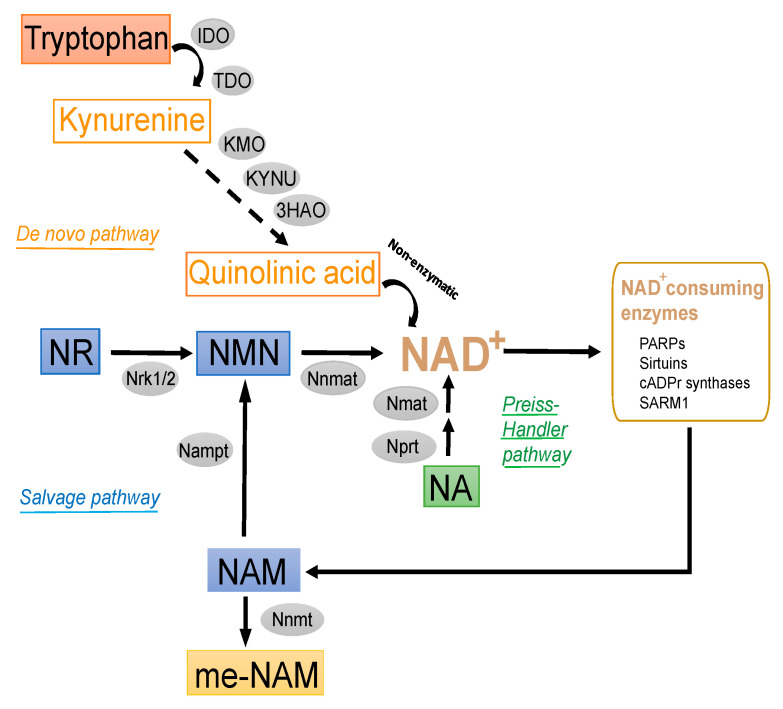
NAD+ precursor metabolism and NAD+-consuming enzymes. Tryptophan, NA, NAM, and NR can be used by different pathways to obtain NAD+. NAD+ formation can be obtained de novo from tryptophan. The first step relies in the conversion of tryptophan to N-formylkynurenine by either indoleamine 2,3-dioxygenase (IDO) or tryptophan 2,3-dioxygenase (TDO). N-formylkynurenine can be converted into quinolinic acid and NAD+ thereafter. NAD+ synthesis from NA is initiated by the NA phosphoribosyltransferase (NPRT), which uses phosphoribosyl pyrophosphate (PRPP) to form NAMN prior to NAD+ synthesis (Preiss-Handler pathway). Finally, the synthesis of NAD+ can be also possible using NAM or NR via the Salvage pathway. This pathway just needs 2 steps for each precursor. NAM is converted by the rate-limiting nicotinamide phosphoribosyltransferase (NAMPT) to form NMN, also using PRPP as a co-substrate. NMN can be also formed by NR phosphorylation through the action of NR kinases (NRK1-2). Finally, the conversion of NMN to NAD+ is eventually catalyzed by cellular NMN adenylyl transferase (NMNAT1-3) enzymes. Abbreviations used were: 3HAO: 3-hydroxyanthranilate 3,4-dioxygenase; IDO: indoleamine 2,3-dioxygenase; KMO: kynureine monooxygenase; KYNU: kynureinase; NAD: nicotinamide adenine dinucleotide; NA: nicotinic acid; NAM: nicotinamide; NMN: nicotinamide mononucleotide; Nampt: nicotinamide phosphoribosyltransferase; Nnmat: nicotinamide mononucleotide adenylyltransferase; NR: nicotinamide riboside; Nrk1/2: NR kinase 1, 2; Nmat: nicotinate mononucleotide adenyltransferase; Nprt: nicotinate phosphoribosyl-transferase; Nnmt: nicotinamide methyltransferase; TDO: tryptophan 2,3-dioxygenase.

Because NAD+-dependent signaling reactions (protein deacetylation by sirtuins, intracellular calcium signaling, and mono-/poly-ADP-ribosylation) involve degradation of the dinucleotide, a constant supply of this molecule is required to reach cellular homeostasis. NAD+ precursors are commonly ingested from dietary sources. Intracellular NAD+ can be technically produced through distinct metabolic pathways (Figure 1): (i) the de novo pathway from tryptophan, (ii) the salvage synthesis pathway from NAM and NR, and (iii) the Preiss-Handler pathway from NA [32,36]. Additionally, intermediate molecules from these NAD+ biosynthetic pathways, such as NMN, can also be direct substrates for NAD+ synthesis [33].

#### 2.1.1. De Novo Synthesis Pathway

The kynurenine pathway drives de novo synthesis of NAD+ from the essential amino acid tryptophan [37] (Figure 1). This metabolic pathway is the dominant route of metabolism for absorbed tryptophan [38], accounting for more than 95% of daily metabolized tryptophan. However, tryptophan is considered an inefficient source of NAD+, as it requires 8 enzymatic reactions and, thus, has lower therapeutic value than other precursors, including NAM, NR, and NMN [39]. Therefore, these last molecules can be considered much more physiologically preferred NAD+ precursors than tryptophan [40]. Although the potential function of the kynurenine pathway of tryptophan metabolism to produce NAD+ has received little attention, emerging evidence suggests potential interplay between this pathway and cardiovascular disease [37,41]. In line with this, the kynurenine pathway has been recently regarded as a potential source of NAD+ in macrophages [42].

#### 2.1.2. Salvage Pathway

NMN is a major intermediate of NAD+ (Figure 1). The salvage synthesis of NMN from NAM is catalyzed by nicotinamide phosphoribosyltransferase (NAMPT), whereas the synthesis of NMN from NR is catalyzed by intracellular NR kinases (NRK1 and NRK2) [43]. Finally, NMN may be subsequently transformed into NAD+ by different subcellular forms of adenylyltransferases (NMNAT1-3) [44].

#### 2.1.3. Other Modulators of Intracellular NAD+ Pools

In addition to these enzymes, others may also influence the tissue abundance of NAD+ (Figure 1). Specifically, nicotinamide N-methyltransferase (NNMT) catalyzes the N-methylation reaction of NAM and other analogous molecules using S-adenosyl methionine (SAM) as a donor of methyl groups [45] and produces me-NAM and S-adenosyl homocysteine (SAH) [39,40]. Recent reports suggest that NNMT may also act as a determinant of tissue NAD+ pools in vivo [43]. Interestingly, the subproduct me-NAM has been reported to exert anti-inflammatory, antioxidant, and antidiabetic effects in different experimental models [46,47,48,49,50].

### 2.2. Administration and Bioavailability of NAD+ Precursors

L-tryptophan can be safely supplemented in the diet [51]. This amino acid can be added directly to feedstuff and dissolved into drinking water [38]. As indicated previously, its availability is primarily governed by the kynurenine pathway, which may contribute to NAM production under dietary deficiency of this vitamin form [38].

NAM is highly soluble in water and is considered a safe food supplement in animal nutrition and the predominant circulating form of vitamin B3 in vivo [52]. Its safety margin is at least ten times the dietary requirements and use levels, and it can be administered orally by feeding or drinking (tap water) routes with similar bioequivalence rates. Oral NAM can be absorbed in the stomach and the small intestine either through passive diffusion or by Na+-dependent carrier-mediated facilitated transport mechanisms [52], with passive diffusion predominating at pharmacological doses. Similarly, NA absorption into the intestinal mucosa follows the same transport mechanisms as NAM. NA can be further metabolized via NA mononucleotide (NAMN) to form the NA analog of NAD+ through the Preiss-Handler pathway (Figure 1). NAD+ can then be enzymatically cleaved into NAM in the intestinal mucosa and then the liver. The latter tissues contain elevated amounts of NAD+-consuming enzymes, such as NAD+ glycohydrolase, that allow NAM synthesis from NAD+. NAM can be subsequently transported to tissues for the synthesis of NAD+ or its phosphorylated form (NADP+). The principal excretory product is the methylated, water-soluble metabolite me-NAM (Subsection: Other modulators of intracellular NAD+ pools) or some of its oxidation products [53].

NR has a good bioavailability [54]. Unlike NA and NAM, dietary NR cell uptake is mediated by a specific nucleoside transporter [55], where it is transformed into NAD+ through a distinct, two-step biosynthetic pathway.

In contrast to that of NR, the bioavailability of NMN is rather limited [56]. Indeed, extracellular sources of NMN do not efficiently cross the endothelial membrane and require previous dephosphorylation by CD73 (ecto-5′-nucleotidase) to generate NR or must be extracellularly metabolized to NAM by CD38 [57]. Interestingly, a specific NMN transporter (i.e., Slc12a8) exists in murine small intestinal epithelium [58]. This transporter is predominantly expressed in the small intestine and in white adipose tissue and the liver to a lesser extent. However, whether Slc12a8 can also be expressed by cells of the vasculature or cardiomyocytes remains to be determined. NMN can also be obtained from NAM by extracellular NAMPT (eNAMPT, also termed visfatin). In endothelial cells, this conversion process requires the participation of phosphoribosyl-1-pyrophosphate (PRPP) [8]. Finally, extracellular NAD+ can be directly transported into cardiomyocytes through connexin 43 (i.e., Cx43) channels [59]. Taken together, these findings suggest that intracellular transport of NAD+ and NMN may be cell-specific and rely on different transporters.

### 2.3. NAD+-Consuming Enzymes as Determinants of the NAD+ Content

In the last two decades, beyond its important contribution to intermediate metabolism, NAD+ has also been shown to influence multiple signaling pathways (reviewed in detail in ref. [8]). Indeed, NAD+ can also be used by different protein regulators, including NAD+-dependent histone deacetylases (also termed sirtuins), which regulate the cellular mitohormetic response and, thus, favorably influence energy metabolism (reviewed in detail in ref. [33]). Moreover, several families of ADP-ribose synthesizing enzymes—mono-(ADP-ribose) polymerases (MARPs) and poly-(ADP-ribose) polymerases (PARPs)—also require NAD+ to specifically catalyze mono-/poly-ADP-ribosylation reactions of target proteins [60]. These enzymes are generally considered DNA damage sensors because of their participation in DNA repair, replication and transcription and the chromatin structure, among other functions [33,34,61,62,63,64]. Other enzymes, such as NAD+-dependent glycohydrolases (i.e., CD38 and CD157) involved in calcium signaling [65,66] and regulation of the cell cycle [66,67,68], use NAD+ to produce the second messenger cyclic-ADP-ribose and ADP-ribosyl cyclase-produced glycoproteins [69].

## 3. In Vitro Cardioprotective Effects of NAD+ Sources

Experimental evidence suggests that oxidative stress and inflammation are mechanistically interconnected in driving cardiac and vascular diseases, and both may contribute to disease progression [70]. Increased vascular reactive oxygen species can react with cellular molecules and organelles, which become dysfunctional, leading to inflammation and endothelial dysfunction with devastating consequences on the vasculature and cardiac tissue. Moreover, impaired vascular reactive oxygen species homeostasis and inflammation may be reciprocally exacerbated, thereby resulting in a vicious and chronic cycle.

Current applications for successful control of enhanced oxidative stress and inflammation for the management of such complex chronic diseases are still elusive, partly due to the extensive diversity of oxidative stress pathways and inflammatory cells that can either be cardioprotective or cause cardiac damage. In this context, we next focus on relevant recent research evidence to examine some cardioprotective properties shown by vitamin B3 forms or intermediates in appropriate cell models of disease.

### 3.1. Ex Vivo Properties of NAD+ Precursors Relevant to Cardiovascular Diseases

Reactive oxygen species, including the superoxide anion (O_2_^•−^) and the nitric oxide radical (NO^•^), are critical bioreactive signaling molecules that may influence both cellular homeostasis and disease. Under physiological conditions, the intracellular pool of such reactive oxygen species is controlled by an intricate array of antioxidant defense systems that include superoxide dismutases, catalase, the glutathione peroxidase/reductase system, and the peroxiredoxin/thioredoxin system [71]. Under pathological conditions, elevated reactive oxygen species can overwhelm cellular antioxidant defenses, impair reactive oxygen species scavenging and, therefore, lead to cardiac dysfunction [72,73,74,75,76].

In addition to the enzymatic antioxidant systems, certain molecules (identified as antioxidants) may serve as direct metabolic scavengers of electrons from reactive radicals and, thus, confer direct protection to biomolecules from oxidative damage by virtue of their chemical ability to accept/donate electrons. In particular, NAM may directly function as an O_2_^•−^ radical scavenger and inhibit the generation of free radicals (e.g., O_2_^•−^, NO^•^, and HCLO^•^) [77] and prevent protein and lipid oxidation in vitro [78]. Importantly, this scavenging ability may paradoxically enable some vitamins with recognized antioxidant properties to function as pro-oxidants under certain circumstances [79,80,81]. Notably and in contrast to other vitamins with antioxidant abilities, such as vitamins A and C, vitamin B3 (NA) did not induce oxidative damage in vitro at physiologically relevant concentrations [82].

**Figure 2:**
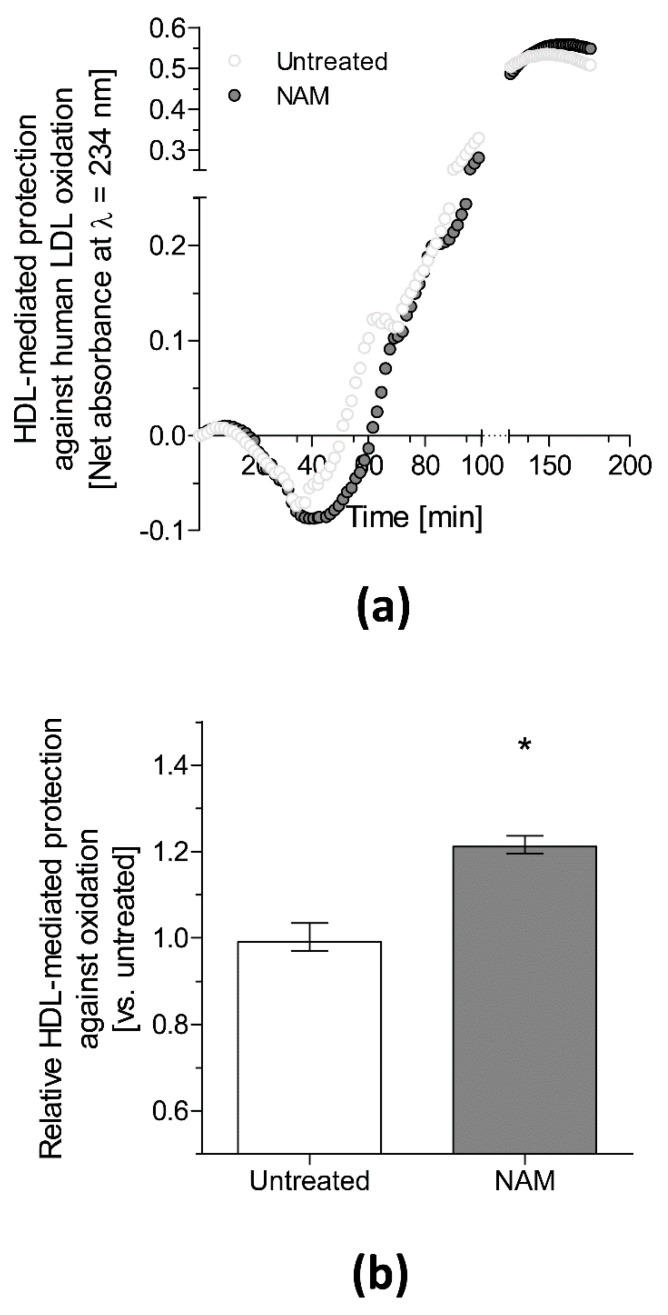
Effect of NAM manipulation on antioxidant ex vivo protection conferred by plasma isolated HDL. (**a**) Representative diene formation curves of human LDL incubated with mouse HDL isolated from untreated and NAM-treated wildtype C57BL/6J mice in the presence of 2.5 μM CuSO_4_ at 37 °C. The final oxidation kinetics of the LDL+HDL mixture was shown after subtracting the kinetics of HDL incubated without LDL. (**b**) HDL antioxidant activity against LDL oxidative modification. Methods used can be found in greater detail in reference [84]. Data were expressed as relative lag phase to LDL oxidized in the presence of HDL isolated from Untreated mice (arbitrary unit = 1). Data were expressed as the median ± interquartile range (*n* = 4–5 independent pools per group). Differences between the mean values were determined using a nonparametric Mann–Whitney test. * indicates significant differences versus untreated mice (*p* < 0.05). Abbreviations used were NAM, NAM-treated mice using 1% NAM dissolved in tap water [85] (Julve et al., 2021, unpublished data).

Both the resistance to oxidation of non-HDL and the antioxidant properties of HDL are two of the most important antiatherogenic metrics of lipoproteins ex vivo [83]. We recently reported that isolated plasma non-HDL from mice lacking apolipoprotein (Apo)E (*Apoe^−/−^*) treated with NAM was less susceptible to oxidation ex vivo than that from untreated mice [25]. Notably, incubation of human low-density lipoprotein (LDL) with NAM produced a direct inhibitory effect on lipid peroxidation during an analysis of the oxidation kinetics of exposed lipoproteins [25]. However, the effect of NAM on the HDL antioxidant capacity of *Apoe^−/−^* mice could not be evaluated in the latter study due to the limited circulating HDL moieties in this mouse model. Instead, the effect was directly assessed using HDL isolated from wild-type mice on the same genetic background as the *Apoe^−/−^* mice. Consistently, our data revealed that HDL isolated from the plasma of NAM-treated mice was more protective against LDL oxidation ex vivo than HDL isolated from untreated mice (Julve et al., 2021, unpublished data, Figure 2), suggesting enhanced atheroprotection by HDL from NAM-treated mice.

### 3.2. Cellular Effects of NAD+-Increasing Strategies Relevant to Cardiovascular Diseases

Compelling experimental evidence suggests a favorable influence of NAD+-restoring strategies on the main cellular mechanisms involved in cardiovascular cell damage.

#### 3.2.1. Oxidation

NAM protects vascular cells against oxidation [78]. This antioxidant property has also been reported for other vitamin B3 derivatives, such as NR [86] and NMN [9], probably suggesting common mechanistic pathways among such different NAD+ precursor intermediates.

The maintenance of intracellular pools of NAD+ is an important vasoprotective mechanism in arterial endothelial cells [86,87]. Consistently, NAD+ depletion is commonly related to age-related vascular dysfunction [88]. Aged endothelial cells exposed to NMN were protected against oxidative stress in a SIRT1-dependent fashion [89]. Similarly, NR-mediated restoration of NAD+ contents in the lung and heart protected against oxidative stress and apoptosis in tissues from an animal model of sepsis [86]. NR exposure prevents reactive oxygen species production and apoptosis in endothelial cells induced by conditioned medium collected from LPS-treated macrophages [86], further supporting the beneficial vascular effects of NR. NR is also protective against alcohol-induced oxidative stress and inflammation in cultured macrophages [90]. Importantly, NR effects occurred via NAD+/SIRT1 signaling [86,90].

More recently, NAD+ depletion in lipopolysaccharide (LPS)-mediated activated macrophages was accompanied by reactive oxygen species-mediated DNA damage and by activation of PARP, an NAD+-consuming enzyme [91]. In this study, the salvage pathway was induced upon NAD+ depletion, as revealed by increased NAMPT expression allowing the maintenance of intracellular pools of NAD+ sufficient for fueling glyceraldehyde-3-phosphate dehydrogenase activity and Warburg metabolism, which is critical for LPS-mediated activation of macrophages [92]. Interestingly, reduced intracellular NAD+ and increased NAMPT expression developed rapidly after inflammatory activation together with DNA damage caused by reactive oxygen species [91].

#### 3.2.2. Inflammation

Insufficient NAD+ leads to vascular inflammation [93]. Accumulating data strongly suggest the contribution of NAD+/SIRT1 signaling to inflammation.

The anti-inflammatory effects of NAM were first described in vitro in multiple immune cell types frequently involved in chronic inflammatory processes, such as atherosclerosis [94,95,96,97,98,99,100,101,102], including activated circulating monocytes and tissue immune cells (i.e., resident macrophages), which are the first inflammatory responders and are ultimately involved in the resolution of inflammation. Notably, NAM can promote the differentiation of monocytes toward macrophages with attenuated inflammatory characteristics, i.e., with strongly reduced proinflammatory features [94]. NA administration may also shift macrophages to M2 polarization in vitro [23]. Similar anti-inflammatory actions in activated macrophages have been reported for NR [90].

Mitochondria play an important role in innate immunity and chronic inflammation [103]. Dysfunctional mitochondria can contribute to the pathophysiology of cardiovascular diseases [104]. Indeed, blockade of de novo synthesis by NAD+ using genetic or pharmacological approaches depleted NAD+ stores, suppressed mitochondrial NAD+-dependent signaling and respiration, and impaired phagocytosis and inflammation resolution [42]. This suggests a critical role for intracellular NAD+ pools in the regulation of macrophage immune function in resting and in aged and immune-challenged macrophages.

Elevated systemic inflammation has been causally linked to mitochondrial dysfunction of peripheral blood mononuclear cells (PBMCs) in subjects with HF, whereas NR-mediated NAD+ elevations improved mitochondrial respiration and attenuated proinflammatory activation of PBMCs isolated from treated volunteers with HF [105].

#### 3.2.3. Apoptosis

Apoptosis may precede cell death and fibrosis and, thus, drive cardiac and vascular dysfunction. Excess reactive oxygen species may damage intracellular proteins, lipids, and DNA and induce apoptotic cell death [106,107]. Under ischemic/hypoxic conditions, NAD+ metabolism is significantly inhibited in myocardial cells and accompanied by reduced cell viability due to enhanced apoptosis [108,109]. Conceivably, strategies to increase NAD+ metabolism may enable cardiomyocytes to evade apoptosis. Supporting this notion, NAM exposure to primary cardiomyocytes and the H9C2 cell line conferred protection against hypoxia-induced apoptosis [108,109,110]. The attenuation of apoptosis alleviated oxidative stress and improved mitochondrial stress in NAM-treated cells [108,109]. Notably, NAM-mediated protection from apoptosis was linked to upregulation of autophagy-related proteins and accompanied by concomitant attenuation of the mTOR pathway in another study [110].

Cardiomyocyte mitochondrial dysfunction and mitochondrial DNA instability are increasingly believed to be important features of the progression of various forms of cardiac disease [111]. Under these circumstances, mitochondrial DNA repair mechanisms are commensurately induced, including PARP activity [112]. Elevations in cellular PARP activity may drain cellular NAD+ reserves [113] and lead to mitochondrial dysfunction and, thus, impaired cardiac function. Conversely, NR-mediated restoration of NAD+ pools improved the mitochondrial unfolded protein response and mitochondrial health in cultured cardiomyocytes subjected to mitochondrial stress conditions [114]. The latter finding suggests that NAM not only confers direct cellular protection against oxidation to vascular cells but also prevents cellular apoptosis during free radical exposure and blocks inflammatory vascular cell activation [115] by preventing membrane phosphatidylserine exposure [116], which is an apoptotic inducer [117]. In line with this assertion, NAD+ depletion is linked to macrophage apoptosis [118], whereas its repletion with NAD+ precursors (NA and NAM) protects cells against apoptosis [86,119].

#### 3.2.4. Mitochondrial Stress

Cellular NAD+ depletion may occur upon increased mitochondrial DNA damage due to prolonged PARP action [113], an NAD+-consuming enzyme involved in mitochondrial DNA repair, which may cause inhibition of other NAD+-dependent proteins, especially sirtuins.

NAM pretreatment attenuated mitochondrial stress and protected hypoxic primary rat cardiomyocytes against cell necrosis and apoptosis [108]. In this study, NAM increased the intracellular ATP content in mitochondria, further revealing that NAM protected cardiomyocytes from hypoxia-mediated mitochondrial damage. Importantly, NAM-mediated favorable effects were accompanied by activation of the AMPK pathway [108]. By increasing the intracellular NAD+ content, NAM can also increase the protein abundance of superoxide dismutase and catalase and induce intracellular energy production [109], thus reducing the abundance of mitochondrial reactive oxygen species and preventing the loss of mitochondrial membrane potential.

#### 3.2.5. Autophagy

Autophagy is a cellular process that recycles components of the cytoplasm of cells and plays a key role in tissue remodeling. As previously mentioned, NAM supplementation protected chronic hypoxic myocardial cells against hypoxia-induced apoptosis and increased the protein abundance of autophagy-related proteins by attenuating mTOR pathways [110].

NR exposure promoted autolysosome generation in neonatal and adult mouse cardiomyocytes exposed to doxorubicin (DOX) [120]. In particular, NR can attenuate autophagic flux blockade, autolysosome accumulation, and oxidative stress in DOX-treated cardiomyocytes. Notably, inhibition of lysosomal acidification or SIRT1 abrogated such NR-mediated protective effects during DOX-induced cardiotoxicity, thereby suggesting that NR enhances autolysosome clearance via NAD+/SIRT1 signaling to prevent DOX-induced cardiotoxicity.

#### 3.2.6. Fibrosis

Organ fibrosis is characterized by excess deposition of extracellular matrix proteins. Administration of NA, NAM, and NR attenuates experimental liver fibrosis [14,121,122,123,124], potentially indicating that an insufficient NAD+ supply may underlie organ fibrosis. Similar favorable effects could be conceivably suggested for NAD+-increasing interventions aiming to directly combat myocardial fibrosis. Although such interventions have not yet been explored, this strategy may warrant further investigation.

## 4. In Vivo Cardiovascular Effects of NAD+-Boosting Strategies: Lessons from Animal Models

### 4.1. Atherosclerosis

Immune cell infiltration and activation are a characteristic finding in chronic inflammation processes, such as atherosclerosis [125]. Macrophages and vascular smooth muscle cells are regulated through a coordinated pivotal program during the development of atherosclerotic lesions [126,127]. Regulation of the cholesterol balance in these cell types is critical in atherogenic processes, and the accumulation of LDL-derived cholesterol in foam cells leads to chronic inflammatory responses in the subendothelial arterial intima [128]. The interaction of LDL with proteoglycans initiates LDL retention in the intima, facilitating LDL modifications and increasing their nonregulated uptake by macrophages [129]. In contrast, HDL stimulates cholesterol efflux from macrophages, the first step of the reverse cholesterol transport pathway, which is mechanistically linked to the anti-inflammatory and immunosuppressive functions of HDL [126].

The first evidence that NA administration reduced atherosclerosis was reported in rabbits [130,131,132,133] and minipigs [134] (Table 1). The antiatherogenic effect of NA was also confirmed in humans [135,136]. Originally, these antiatherogenic effects were mainly attributed to the ability of NA to reduce serum LDL cholesterol (LDL-C) and triglycerides while increasing HDL-C levels. However, these antiatherogenic effects did not always correlate with NA-mediated hypolipidemic effects [130,134]. The development of genetically engineered mice permitted us to evaluate the potential of NA to prevent massive atherosclerosis in different mouse models of hyperlipidemia and investigate the potential atheroprotective mechanisms. NA had a strong lipid-independent antiatherogenic effect on LDL receptor-deficient (*Ldlr*^−/−^) mice, which was mainly mediated by its receptor GPR109A on bone marrow–derived cells [137]. GPR109A activation by NA enhanced ATP binding cassette (ABC) G1-dependent macrophage cholesterol efflux to HDL, inhibited MCP-1–induced macrophage recruitment into the peritoneal cavity, and impaired macrophage homing into atherosclerotic lesions [137]. Furthermore, NA administration in *Apoe^−/−^* mice also reduced advanced lesion progression and plaque inflammation independent of plasma lipoprotein levels, rendering advanced atherosclerotic lesions more stable [138]. In line with these findings, an independent study also showed that both *Ldlr*^−/−^ and *Apoe^−/−^* mice treated with NA had significantly lowered aortic cholesterol levels, although this study also reported an NA-mediated hypolipidemic effect [139]. However, these findings were not reproduced in another study conducted in *Apoe^−/−^* mice [140], likely because the researchers used a low-fat diet combined with an insufficient NA dose and a short treatment period. On the other hand, *Apoe3*Leiden transgenic mice overexpressing human cholesteryl ester transfer protein (CETP) treated with NA also showed reduced atherosclerosis development concomitant with enhanced very-low density lipoprotein clearance and low CETP activity resulting in higher HDL-C [141]. In this study, NA also reduced monocyte adhesion and macrophage homing into atherosclerotic lesions but promoted macrophage-to-feces reverse cholesterol transport [141], thereby emphasizing that multiple mechanisms may explain the antiatherogenic effects of NA in vivo.

Other vitamin B3 intermediates, such as NMN and NR, reversed age-related endothelial dysfunction and reduced oxidative stress [15,28]. Although their mechanism of action in atherosclerosis prevention has not been directly evaluated in vivo, the effects of these intermediates can be at least partly explained by their ability to effectively increase the tissue content of NAD+ and, thus, restore SIRT1 activity (reviewed in reference [8]). Supporting this assertion, a host of studies in genetically engineered atherosclerotic mice suggest a protective role of SIRT1, although this effect was mainly related to its expression in vascular cells (reviewed in Reference [142]). However, the potential involvement of a SIRT1-mediated mechanism was reflected only by the upregulation of some aortic targets of liver X receptor (LXR) [143]. Supporting this notion, our group has demonstrated that the gene expression of *Abca1* and *Abcg1* was upregulated in aortic tissue from *Apoe^−/−^* mice and in cultured macrophages exposed to NAM, although only marginally [25]. In the latter study, dietary NAM supplementation prevented aortic atherosclerosis in *Apoe^−/−^* mice, which was related to an improvement in the susceptibility of ApoB-containing lipoproteins to oxidation and to upregulation of aortic IL-10 and downregulation of TNFα, suggesting a switch toward anti-inflammatory macrophages [25]. Rather surprisingly, these antiatherogenic effects were accompanied by higher levels of plasma ApoB-containing lipoproteins and impaired macrophage-specific reverse cholesterol transport in vivo [25,144]. Overall, these findings support that NAM-mediated anti-inflammatory effects may prevail in atherogenesis. In line with these findings, me-NAM administration, a major endogenous metabolite of NAM (Section 2), also prevents atherosclerotic plaque progression and inflammation in double *Ldlr^−/−^/Apoe^−/−^* mice [29]. In this study, me-NAM improved endothelial function [29], as was also reported in an independent study conducted in *Apoe^−/−^* mice [30].

NAMPT is the rate-limiting enzyme in the NAD+ salvage pathway. However, different studies investigating the effects of NAMPT in atherosclerosis in vivo found conflicting results depending on the localization of NAMPT. Administration of the NAMPT inhibitor FK866 reduced inflammation in atherosclerotic plaques after carotid cast implantation in *Apoe^−/−^* mice by reducing CXCL1-mediated activities on neutrophils and improving plaque vulnerability parameters [145]. Additionally, liver-specific deficiency of NAMPT increased plasma HDL-C levels and reduced aortic atherosclerosis, including macrophage homing [146]. These effects were closely related to upregulation of LXR target genes and a consequently enhanced macrophage-to-feces reverse cholesterol transport rate in vivo [146]. Conversely, NAMPT overexpression aggravated atherosclerotic inflammation and enhanced atherosclerosis development in *Apoe^−/−^* mice [147]. In contrast, hematopoietic overexpression of human NAMPT attenuated the plaque burden and stabilized lesions in *Ldlr^−/−^* mice [148], likely suggesting tissue-specific and possibly species-specific differences to explain such apparently divergent findings. Notably, these effects were found only after modulating intracellular levels of NAMPT and were associated with macrophage resistance to apoptosis, attenuating monocyte intravasation and migration in response to chemotactic signals, thereby skewing monocyte differentiation and macrophage polarization toward an anti-inflammatory M2 phenotype [148].

Finally, NAD+ is also a substrate for poly(ADP-ribose) polymerase, also known as PARP (Section 2). PARP activity leads to NAD+ and ATP depletion after various types of DNA damage [149]. Both PARP pharmacological inhibition and gene deletion reduced plaque sizes in *Apoe^−/−^* mice concomitant with reduced macrophage homing in two independent studies [150,151]. In particular, PARP inhibition increased plaque stability without improving the lipoprotein profile. Consistently, PARP inhibition also reduced traits of plaque disruption in a separate study conducted in *Apoe^−/−^* mice [152], mainly by decreasing oxidative DNA damage and iNOS-associated protein nitration. In two of these studies, *Parp* gene deletion protected against oxidant-induced foam cell death [150,152], which may be related to preserved intracellular NAD+ and ATP pools. Moreover, pharmacological inhibition of PARP action also promoted atherosclerotic plaque regression in this mouse model of atherosclerosis by modulating most of these key antiatherogenic factors [153]. Consistent with these findings, a recent report confirmed the in vivo antiatherogenic effects of PARP inhibition in rabbits [154], which were likely due to its ability to improve endothelial function by preserving NAD+ levels.

### 4.2. Ischemic-Infarcted Myocardium or Ischemia-Reperfusion

Myocardial infarction is a common major cardiovascular event that originates from myocardial ischemia in the presence/absence of reperfusion [155]. The term ischemia encompasses a broad range of clinical hypoxia scenarios ranging from angina to permanent occlusion. Although reperfusion intervention is clinically mandatory to treat ischemic injury, the associated cardiac damage caused by this intervention should not be overlooked.

The loss of several dinucleotide species, including NAD+, NADH, NADP+, and NADPH, has been shown in myocardial tissue after ligature of the coronary artery branch [156] in dogs. In the corresponding study, NAD+ decline was accompanied by increased activity of NAD+ glycohydrolase, an NAD+-consuming enzyme (Section 2), which has been reported in response to acute experimental acute infarction [157]. Notably, irreversible postischemic injury occurred when NAD+ depletion was approximately 60–70% [158]. Therefore, NAD+ stabilization appears to be critical for the recovery of mitochondrial and cardiac function. NAD+ mainly acts by reducing oxidative stress and cell death [159]. Importantly, cardiac-specific overexpression of NAMPT increased NAD+ levels in the heart and reduced the size of myocardial infarction in response to prolonged ischemia/reperfusion in mice [160]. In this context, NAD+ supplementation also protected hearts from ischemia/reperfusion injury in mice [161], Bama miniature pigs [162], and rats [159,163] (Table 1). Furthermore, cardiac hypoxia-ischemia injury can be relieved in mice treated with NMN supplementation [164]. NMN also protected against ischemia/reperfusion injury in aged rats [165]. NMN replenished heart NAD+ levels but reduced oxidative stress and mitochondrial ROS levels. Another study also suggested that NRK-2 alleviates ischemia-induced HF through P38 signaling [166]. Finally, NR was also shown to be effective in improving vascular function. NR improved endothelium-dependent relaxation of isolated rat mesenteric arteries in an ischemia-reperfusion model [167]. Overall, these studies strongly suggested that NAD+ and its precursors improve alterations following ischemia/reperfusion injury in vivo.

### 4.3. Cardiomyopathy and Heart Failure (HF)

Cardiomyopathy is one of many causes of HF. Its prevalence continues to rise over time, especially with aging.

Dilated cardiomyopathy is characterized by dilatation of the left and right ventricles, leading to systolic dysfunction and a reduced ejection fraction [168]. Susceptibility to dilated cardiomyopathy has been predominantly explained by genetic mechanisms, and, in Western societies, this cardiac condition is the principal indication for heart transplantation.

Various studies have explored HF and NAD+ levels in vivo. Cellular NAD+ is a key regulator of metabolism and bioenergetics, and failure of cardiomyocyte bioenergetics is a critical hallmark of HF. Interestingly, previous studies suggest that mitochondrial dysfunction and metabolic remodeling in HF are associated with an imbalance in the intracellular ratio of NADH to oxidized NAD+ or the NADH/NAD ratio [169,170]. Therefore, controlling intracellular NAD+ levels may improve myocardial bioenergetics and cardiac function. In fact, in a recent paper, normalization of NADH/NAD through supplementation with NAD+ precursors was shown to be associated with cardiac function improvement in an HF mouse model [171]. Specifically, the authors studied the impact of NAD+ precursor supplementation in food (NR) in two different mouse models of dilated cardiomyopathy and found stable myocardial NAD+ levels in the failing heart. Diguet et al. [171] proposed that the benefit observed in their study was due to improved energy metabolism rather than altered protein acetylation. However, previous studies found that NAD+ availability was associated with increased protein acetylation in the failing myocardium, which was attributable to impaired NAD+-dependent protein deacetylation by sirtuins, especially SIRT3 [172,173,174,175]. Another study reported oral supplementation of NR to treat cardiomyopathy caused by laminin A/C gene (*Lmna*) mutations (LMNA cardiomyopathy) in mice. Mutant mice suffered from dilated cardiomyopathy [176]. The NAD+ salvage pathway has been shown to be altered in the hearts of mice and humans carrying *Lmna* mutations, leading to alteration of PARP-1, which is an NAD+ cosubstrate enzyme [177]. Oral administration of NR to *Lmna*^H222P/H222P^ mice resulted in an improvement in the NAD+ cellular content, an increase in PARylation of cardiac proteins, and an improvement in left ventricular structure and function [177]. However, NAM supplementation to *Lmna*^H222P/H222P^ mice did not change NAD+ contents in the liver or heart or cardiac left ventricular function [177].

In general, approximately half of subjects with HF present with a reduced ejection fraction (HFrEF), and the other half present with a preserved ejection fraction (HEpEF) [178], which is important to emphasize because these presentations reflect different but not entirely distinct pathogenic mechanisms, and their treatments may also be different. For example, mitochondrial dysfunction and metabolic remodeling are known to play important roles in HFrEF, but little is known about the mechanisms of mitochondrial dysfunction in HFpEF. In the myocardium of an HFpEF mouse model, mitochondrial fatty acid oxidation has recently been observed to be impaired and to be associated with hyperacetylation of key enzymes along the pathway. SIRT3 downregulation and NAD+ deficiency secondary to an impaired NAD+ salvage pathway are the main contributors to mitochondrial fatty acid oxidation according to the authors of Reference [179]. Interestingly, supplementation with NAD+ or a direct activator of NAD+ biosynthesis in HFpEF mice with NR ameliorated the HFpEF phenotype by improving mitochondrial function and pathological cardiac remodeling [179].

In the setting of aging, the role of NAD+ has been explored by Tannous et al., who demonstrated that a lack of NRK2 does not influence the NAD+ level in the myocardium in young adults [180]. However, NRK2 expression is required to support the maintenance of NAD+ levels in 24-month-old mouse hearts. At this late stage, the major biosynthetic pathways are repressed, and, in the absence of myocardial NRK2, NAD+ levels fall by 50% [180]. Moreover, *Nmrk2*-deficient mice, which develop a progressive DCM-like phenotype with aging, are viable but develop progressive cardiac dysfunction and eccentric cardiac remodeling associated with an alteration in laminin deposition in the extracellular matrix starting at 4 months, followed by cardiac fibrosis and structural defects in the adult myocardium [180]. Consistent with these findings, another study suggested that feeding aging mice with NR improved mitochondrial function and preservation of stem cell function, which may contribute to the extended lifespan of NR-treated mice [11]. Additionally, iron overload and iron deficiency have been associated with cardiomyopathy and HF. Inactivation of transferrin receptor (*Tfr1*) in mice was associated with cardiomegaly, poor cardiac function, failure of mitochondrial respiration and mortality in the second week of life. Interestingly, the lifespan of *Tfr1*-deficient mice was prolonged by treatment with NR [181]. However, the benefit and safety of NAD+ precursor supplementation require further elucidation. For instance, in another study with a high-dose NR supplementation in a cardiomyocyte mutUNG1 mouse model, which is a mouse model with myocardial mitochondrial DNA damage, sirtuin activity was inhibited rather than increased due to intracellular NAM accumulation, while the addition of NR failed to improve cardiac function [182]. Thus, the authors suggested that high doses of NR should be used with caution, especially when cardiomyopathic symptoms are caused by mitochondrial dysfunction and mitochondrial DNA instability.

As stated above (Section 2), NAMPT, a rate-limiting enzyme in the NAD+ salvage pathway, plays an important role in controlling the level of NAD+. In another mouse model of HF, Byun et al. demonstrated that pressure overload-induced HF is exacerbated in both systemic *Nampt*-deficient mice and mice with cardiac-specific *Nampt* overexpression. Unexpectedly, both loss- and gain-of-function models exhibited reduced protein acetylation, suppression of metabolic genes, and mitochondrial energetic dysfunction. Therefore, endogenous NAMPT plays a protective role under pressure overload conditions, but cardiac-specific *Nampt* overexpression is detrimental rather than therapeutic. The authors suggested that these deleterious effects in NAMPT transgenic mice may be partially due to SIRT1 and suppression of the gene expression of key metabolic targets [183].

Another pathophysiological process promoting HF progression is cardiac fibrosis, which is characterized by increasing ventricular stiffness and a reduced cardiac pumping capacity. In a mouse model of isoproterenol-induced cardiac fibrosis, Wu et al. demonstrated that NMN treatment attenuates cardiac fibrosis in vivo and fibroblast activation in vitro by suppressing oxidative stress and SMAD3 acetylation in an NAD+/SIRT1-dependent fashion [184].

Diabetic cardiomyopathy is another major cause of HF. Subjects with diabetic cardiomyopathy have adverse structural remodeling due to enhanced cardiac hypertrophy and fibrosis, resulting in characteristic cardiac dysfunction. The prevalence of diabetic cardiomyopathy is increasing in parallel with the increase in diabetes mellitus. Accordingly, epidemiological studies show a positive correlation of hyperglycemia with an increase in HF in subjects with either type 1 or type 2 diabetes mellitus. Notably, the manifestation of these conditions is independent of atherosclerotic cardiovascular disease and hypertension [185,186,187]. Additionally, hyperglycemia has been shown to be associated with a reduced NAD+ content, a decreased NAD+/NADH ratio, and promoted protein hyperacetylation and mitochondrial dysfunction in diabetic organs [188,189,190,191,192,193,194]; thus, NAD+ restoration is of therapeutic importance. More recently, NAD+ redox imbalance has been shown to alter protein acetylation and phosphorylation in two different mouse models of diabetic cardiomyopathy [195]. Specifically, an NAD+ redox imbalance in mice with cardiac-specific knockout of complex I subunit *Ndufs4* (cKO) hearts causes systolic and diastolic dysfunction due to cardiomyocyte dysfunction. Moreover, diabetic cKO hearts presented increased acetylation of superoxide dismutase 2, protein oxidation, troponin I S150 phosphorylation and impaired energetics. Importantly, all these effects were normalized when cardiac NAD+ levels were elevated using *Nampt* transgenic mice (diabetic cKO-NAMPT mice), suggesting a beneficial effect of exploiting NAD+ metabolism for diabetic cardiomyopathy. Consistent with this finding, restoration of cardiac NAD+ with NMN supplementation in the myocardium of genetically deficient mice with cardiac-specific expression of Kruppel-like factor 4 (*Klf4*) (CMK4KO) protected against pressure overload-induced HF induced by transverse aortic constriction [194]. Notably, CMK4KO mice are more prone to pressure overload-induced HF caused by transverse aortic constriction [196,197]. KLF4 belongs to a larger family of zinc-finger transcription factors that regulate critical cellular processes, such as cell survival, apoptosis, and metabolism. Some members of the KLF family influence several cell types belonging to the cardiovascular system [198]. Specifically, KLF4 is a critical regulator of cardiac mitochondrial homeostasis [197]. Conceivably, its cardiac deficiency was associated with cardiac NAD+ deficiency and mitochondrial protein hyperacetylation coupled with reduced SIRT3 in the hearts of mice [198]. Notably, cardiac NAD+ replenishment with NMN supplementation successfully protected these mice from cardiac disease by positively influencing mitochondrial health and oxidative stress and preventing cardiac cell death in treated mice.

Cardiac hypertrophy is also a common feature in many cardiovascular diseases [199]. Although cardiac hypertrophy may develop as a benign, adaptive response to either physiological or pathological situations, chronic cardiac stimulation can cause cardiac hypertrophy, remodeling and dysfunction and eventually lead to HF [200]. Enhanced inflammation via activation of the nucleotide-binding and leucine-rich repeat pyrin domains protein 3 (NLRP3) inflammasome and concomitant secretion of cytokines contributes to the progression of cardiac hypertrophy [201]. Therefore, cardiac hypertrophy inhibition has been considered a possible target for novel therapies. In this context, a recent study demonstrated that NR administration favorably influenced cardiac hypertrophy and dysfunction induced by transverse aortic constriction (TAC) surgery in a mouse model by directly inhibiting myocardial NLRP3 inflammasome activation [202]. Notably, this NR-mediated NAD+ rise in myocardial tissue elevated SIRT3 activity, which resulted in MnSOD deacetylation and TAC-induced myocardial oxidative stress alleviation.

### 4.4. Cardiotoxicity

Cardiotoxicity can occur during cancer treatment and can reduce quality of life and increase the risk of death from cardiac-related causes [203,204]. This condition can appear within days, months, or years after chemotherapy, even after disease remission, which is the case, for example, for doxorubicin (DOX), a drug widely used in chemotherapy for the treatment of several malignancies. Nonetheless, the dose-dependent cardiotoxic effects of DOX, which include increased cardiomyocyte death via apoptosis [205,206], necroptosis [207] and ferroptosis [208], left ventricular dysfunction, dilated cardiomyopathy, and congestive HF in severe cases [209], limit its use in clinical practice; therefore, intensive research to attenuate such adverse effects is currently underway. Importantly, oxidative stress is commonly increased upon DOX treatment and underlies cardiotoxicity. In this context, NAM administration to DOX-treated rats successfully reversed signs of cardiac tissue injury revealed by elevated serum cardiotoxicity molecules and conduction and histopathological abnormalities [210]. Consistently, NR also reduced cardiac injury and myocardial dysfunction in DOX-treated mice by restoring NAD+ levels [120].

### 4.5. Aortic Aneurysm: Vascular Disease

Aortic diseases can be extremely life-threatening and include a variety of clinical entities, the most frequent of which is aortic aneurysm. Aortic aneurysm (AA) refers to progressive localized dilatation and weakening of the aorta, which can occur in the chest, thorax (thoracic aneurysm, TAA) or abdomen (abdominal aortic aneurysm, AAA). Although differences can be found in the pathobiology between TAA and AAA, both are characterized by local chronic inflammation of the aortic wall, apoptosis of smooth muscle cells in the aortic media layer and fragmentation of the extracellular matrix of the aorta at the site of the aneurysm. Increased local expression of proinflammatory cytokines, extracellular matrix degradation, microcalcification, and oxidative stress are biological processes contributing to degeneration of the aorta [211].

The risk factors for developing aortic aneurysms are age, sex, smoking, high blood pressure, peripheral artery disease, genetic disorders (individuals with Marfan, Ehlers–Danlos, Loeys–Dietz, or Turner syndrome can develop TAA), a family history of aneurysmal diseases, inflammatory diseases of the blood vessels (vasculitis), and aortic valve abnormalities. No effective drug therapy is currently available for AA, and 70% of small AAs grow to a size requiring surgical repair before fatal aortic rupture occurs [211]. Accordingly, identifying targets for the development of drug therapies that can effectively limit AA growth and rupture has attracted strong interest.

The sequential pathophysiology of aneurysm formation is unclear, but vascular smooth muscle cells (VSMCs) are known to play a central role in vascular cell vitality and, thus, matrix integrity. NAMPT is expressed in cultured SMCs, and its content and activity decline during advanced smooth muscle cells (SMC) aging [212,213,214]. Knockdown of *Nampt* in VSMCs has been shown to increase VSMC apoptosis, which has an important influence on AA development [215], whereas overexpression of *Nampt* can enhance VSMC survival [212], thereby suggesting a potential contribution of the salvage pathway to NAD+ synthesis in this cell type. Although controversial, some reports show that the NAMPT-NAD+ axis control system may favorably influence SMC contractile function [216,217,218].

NAMPT is expressed in periaortic adipose tissue [219], and altering NAD+ metabolism through the diet has been found to suppress age-related aortic dysfunction [220]. Watson and colleagues showed that the aortas of patients with thoracic aortic aneurysms had lower levels of NAMPT than nondilated aortas and correspondingly increased DNA strand breakage in SMCs [217]. They generated mice with homozygous deficiency of NAMPT in SMCs and found that loss of NAMPT rendered the aorta susceptible to aortic dilation and rupture and that their SMCs were more prone to senescence and DNA damage. Finally, they found that the NAMPT promoter was hypermethylated in the aortic media of aortopathy patients, suggesting epigenetically repressed NAMPT production. Together, the results uncovered an endogenous NAD+-fueling system in the aortic media that protects against DNA damage and premature SMC senescence.

The pharmacologic potential of NR in TAAs associated with genetic disorders has been investigated [221]. Using a mouse model with specific deletion of mitochondrial transcription factor A (*Tfam*) in VSMCs, *Tfam*-deficient VSMCs were observed to acquire a senescent and proinflammatory phenotype together with impairment in their contractile function [221]. These mice develop aortic aneurysms, medial degeneration, and lethal dissections, and NR supplementation rapidly raised TFAM levels, improved mitochondrial metabolism, and normalized aortic function and diameter in the Marfan mouse model of TAA.

NA inhibits vascular inflammation by decreasing oxidative stress and inhibiting monocyte/macrophage inflammatory responses and inflammatory cytokine production, which are key events in the pathogenesis of AAA [222,223]. Accordingly, NA may of particular interest for the prevention and treatment of aortic aneurysms [224,225].

In this regard, a previous study using two distinct animal models of AAA reported that NA inhibited AAA formation in conjunction with reduced aortic immune cell infiltration, inflammation, and matrix degradation [24]. The beneficial effects of NA are independent of GPR109A signaling, similar to NAM, and likely mediated through NAD+ production leading to SIRT1 activation. These results suggest that boosting NAD+ levels and SIRT1 activity by supplementation with NAM or related biomolecules may represent a promising therapeutic strategy for AAA treatment and prevention [24].

The role of SIRT1 in vascular homeostasis and diseases in mammals has been well documented [226,227,228]. Enhancing NAD+ biosynthesis with NAD+ precursors, such as NMN and NR, increases the activity of SIRT1 [10,12,229,230], improving mitochondrial dysfunction in different pathologies [33,231,232]. Notably, supplementation with NMN restored arterial SIRT1 activity and reversed age-associated arterial dysfunction and oxidative stress in the aortas of aged mice, which was associated with partial normalization of structural proteins in the arterial wall [9]. Additionally, acute NMN incubation in isolated aortas increased NAD+ threefold, supporting the promising potential of NMN supplementation for the treatment of arterial aging [9]. This is consistent with previous reports showing that NMN increases NAD+ in cultured vascular endothelial cells [233] and normalizes the ratio of NAD+/NADH in aortic tissue [234].

Downregulation of SIRT1 might also be mechanistically linked to AAA pathogenesis. Compared with those in adjacent control sections, SIRT1 expression and activity are significantly decreased in human AAA lesions [235]. In *Apoe^−/−^* mice infused with Ang II using pumps to induce hypertension (a recognized animal model of aneurysm), the incidence of AAA and the mortality rate were markedly reduced in VSMC-specific, *Sirt1* transgenic mice [235]. Even without *Apoe* deficiency, an increase in both the prevalence and mortality of AAAs was found in VSMC-specific *Sirt1* deficient mice compared with wild-type mice. Similar data were obtained in a calcium chloride–induced mouse AAA model in independent studies [235]. The underlying mechanism involves attenuation of vascular senescence by SIRT1 via modulation of the p53/p21 pathway. Moreover, macrophage-specific *Sirt1* expression was reported to contribute to AAA protection [236]. Reducing calorie intake protected against AAA formation in *Apoe^−/−^* mice by upregulating the expression and activity of SIRT1 in VSMCs [237], and specific knockout of VSMC-derived *Sirt1* abolished the prevention of AAA by caloric restriction [235].

In addition to AAAs, SIRT1 is also important in the prevention of TAA. VSMC-specific *Sirt1* deficient mice did not influence the incidence of TAA but increased the fatality rate in mice. Moreover, VSMC-specific *Sirt1* overexpression markedly blocked TAA development mainly through epigenetic downregulation of MMP-2 [238].

These findings provide evidence that SIRT1 reduction links vascular senescence through vascular inflammation to AAAs and that SIRT1 in VSMCs is a promising therapeutic target for the prevention of life-threatening aortic diseases.

Finally, CD38, another NAD+-consuming enzyme, is a key modulator of NAD+ metabolism and influences cell signaling, aging, and tumor biology, suggesting a role for this enzyme as a target with promising therapeutic potential. CD38 was found to be highly expressed in aortic aneurysm tissue, and circulating CD38 was positively correlated with peak wall stress values, but the mechanisms by which CD38 was involved in AAA remain unclear [239].

### 4.6. Hypertension

Hypertension, also known as high or increased blood pressure, is a condition in which blood vessels are subjected to persistently elevated pressure, eventually causing health problems, such as heart disease [240]. The pathogenesis of essential hypertension is complex and likely multifactorial, involving genetic factors, activation of neurohormonal systems, such as the sympathetic nervous system and renin-angiotensin-aldosterone system, obesity, and increased dietary salt intake. This entity involves the interaction of multiple organs and numerous mechanisms, with the kidney playing a central role, as it is both the contributing and the target organ of the hypertensive processes. In accordance with most major guidelines, a subject with hypertension is defined by a systolic blood pressure (SBP) ≥ 140 mm Hg and/or a diastolic blood pressure (DBP) ≥ 90 mm Hg on repeated examination [241].

Studies exploring NAD+ and hypertension relationships in vivo have shown that endothelial dysfunction by SIRT impairment is involved in vascular homeostasis and cardiovascular disease and is related to hypertension [242]. Several studies in hypertensive mice described that, under the pathological stress of hypertension, endothelial-specific deletion of *Sirt6* further exacerbated endothelial dysfunction and cardiorenal injuries, indicating the importance of SIRT6 in maintaining endothelial functions and preventing hypertension and its complications by ensuring endothelial function [242,243]. Inhibition of angiotensin II-activated TGF-β by SIRT1 has been described to contribute to the improvement of vascular remodeling (including reduced vascular inflammation and collagen synthesis) in the aortas of transgenic mice, resulting in a decreased SBP [244]. Other in vivo studies have also demonstrated the vasorelaxing capacity of NAM through inhibition of ADP ribosyl cyclase (a mediator of renal vasoconstriction in vivo through the Ca^2+^ signaling pathway) [245,246,247] (Table 1). In a previous study performed in spontaneously hypertensive rats receiving NADH, a lower SPB in treated rats versus nontreated rats was observed after 60 days of treatment [248]. In an in vivo study in rats with renal mass reduction, long-term NA supplementation ameliorated hypertension and partially reversed the upregulation of oxidative, inflammatory, and profibrotic mediators in the remnant kidney [249]. NAM has also been used to treat preeclampsia (high blood pressure and proteinuria in pregnant women) in a reduced uterine perfusion pressure mouse model, which was found to be effective in improving and preventing hypertension, fetal growth restriction and premature birth [250]. In a similar study involving 2 mouse models of preeclampsia, the authors concluded that NAM decreased blood pressure and endotheliosis in the mothers, probably by inhibiting ADP ribosyl cyclase, and also prevented fetal growth restriction, probably by normalizing fetal ATP synthesis via the nucleotide salvage pathway [251]. The same group also reported that a pharmacological dose of NAM normalized blood pressure in mice with impaired eNOS function either pharmacologically or genetically by suppressing inflammation and had partial benefits on kidney damage induced by L-NAME [252]. A previous work carried out in C57BL/6J mice demonstrated that upregulation of monocyte-derived extracellular NAMPT contributed to preservation of myocardial NAD+ levels and functional compensation against pressure overload, which is crucial to avoid HF [253]. In a recent study regarding HF with a HFpEF, oral supplementation of NAD+ precursors, such as NAM, in Dahl salt-sensitive rats, improved hypertension and diastolic dysfunction, and these effects were mediated partly through alleviated systemic comorbidities, enhanced myocardial bioenergetics and ameliorated cardiomyocyte passive stiffness, and calcium-dependent active relaxation [31].

Nonetheless, most authors conclude that the mechanisms underlying the observed effects explaining how NAD+ precursors reduce blood pressure are still unclear. NAD+-consuming enzymes, ADP ribosyl cyclase and SIRTs, and the anti-inflammatory effects of NAD+ have been hypothesized to contribute to blood pressure reduction; however, this assertion requires further research [6].

**Table 1:** In vivo evidence for NAD+-increasing strategies in different animal models of different cardiovascular pathologies.

Form of Suppl. B3	Animal Model	Dose and Route of Administration	Duration	Outcome	Reference
**Atherosclerosis**					
NA	*Ldlr^−/−^* mice	Dietary supplementation of NA (0.3% *w*/*w*) in a high-fat diet containing 21% butter fat and 1.5% cholesterol	10 weeks	Reduced aortic atherosclerotic plaque area	[137]
NA	*Apoe^−/−^* mice	Dietary supplementation NA (0.5% *w*/*w*) in a regular chow diet	27 weeks	Reduced atherosclerotic lesions within the innominate artery	[138]
NA	*Apoe^−/−^* mice and *Ldlr^−/−^* mice	Dietary supplementation of NA (3% *w*/*w*) in a high-fat diet containing 21% of fat and 0.2% cholesterol	8 weeks	Reduced aortic cholesterol and whole atherosclerotic plaque area	[139]
NA	*Apoe^−/−^* mice	Dietary supplementation NA (0.5% *w*/*w*) in a regular chow diet containing 0.2% cholesterol	14 weeks	No changes in aortic root atherosclerotic area	[140]
NA	Apoe*3Leiden transgenic mice expressing human CETP	Dietary supplementation NA (0.1% *w*/*w*) in high fat diet containing 15% cacao butter and 0.1% cholesterol	18 weeks	Reduced aortic root atherosclerotic area	[141]
NA and pentaerythritoltetran NA	Rabbit	Dietary supplementation of NA or pentaerythritoltetran NA (0.5% *w*/*w*) in a cholesterol-containing (1%) diet	71 days	Only pentaerythritoltetran NA reduced the lipid infiltrated area of the aorta	[132]
NA and pentaerythritoltetran NA	Rabbit	Dietary supplementation of NA or pentaerythritoltetran NA (0.5% *w*/*w*) in a cholesterol- and coconut oil- containing (1% and 3%, respectively) diet	81 days	Both drugs reduced the lipid infiltrated area of the aorta	[132]
Pentaerythritoltetran NA	Rabbit	Dietary supplementation of pentaerythritoltetran NA (0.5% *w*/*w*) in a coconut oil- containing (8% and 15%) diet	120 and 160 days	Reduced lipid infiltration in the aorta	[131]
Pentaerythritoltetran NA	Rabbit	Dietary supplementation of pentaerythritoltetran NA (0.75% *w*/*w*) in a coconut oil- containing (3%) diet. The concentration of cholesterol in the diet was adjusted for each rabbit andt the animals attained a mean plasma cholesterol level of about 6 mg/mL	160 days	Reduced aortic cholesterol	[130]
NA and Pentaerythritoltetran NA	Mini-pigs	Dietary supplementation of NA or pentaerythritoltetran NA (0.25–0.75% *w*/*w*) in egg yolk- and cholesterol- (11 and 0.5–0.75%, respectively) containing diet	12–19 months	Reduced lipid infiltration in the aorta	[134]
Me-NAM and NA	Double *Ldlr^−/−^/Apoe^−/−^* mice	Administration of me-NAM or NA (0.1 g/kg/day) in the drinking water together a regular chow diet	4 weeks	Both drugs reduced aortic root atherosclerotic area	[29]
NAM	*Apoe^−/−^* mice	Administration of NAM (0.25 and 1% *w/v* equivalent to 0.5 and 1.9 g/kg/day, respectively) in the drinking water together a high-fat diet containing 21% of fat and 0.2% cholesterol	4 weeks	Reduced aortic root atherosclerotic area	[25]
me-NAM	*Apoe^−/−^* mice	Dietary supplementation of me-NAM (0.0057 and 0.017% *w*/*w*) in a high-fat diet containing 21% of fat and 0.2% cholesterol	8 weeks	Reduced aortic root atherosclerotic area	[30]
**Cardiotoxicity**					
NAM	Sprague Dawley male rats treated with DOX (5 mg/kg, i.p.) once/week for four consecutive weeks	Oral dose of NAM (600 mg/kg by oral gavage).	28 consecutive days	Amelioration of cardiotoxic serum cardiotoxicity indices, conduction and histopathological abnormalities	[210]
NR	Male mice (aged 2 months) were injected with a single dose of DOX (20 mg/kg, i.p.)	0, 100, 300, or 500 mg/kg (i.p.) given 30 min prior DOX injection	5 days	Reduced cardiac injury and myocardial dysfunction	[120]
**Myocardial ischemia/reperfusion injury**					
NAD+	Male Wistar rats	10–20 mg/kg intravenous (i.v) (approximately 85% reduction of the infarct at the dosage of 20 mg/kg)	A single dose immendiately before ischemia The rats were sacrificed 6 and 24 h after reperfusion.	Reduced the infarct size after ischemia/reperfusion Attenuated apoptotic damage and enhancing the antioxidant capacity	[159]
NAD+	C57BL/6 wild-type mice	i.p. administration of 0.2 g/kg NAD	1 dose before myocardial injury	Reduced myocardial infarct size after ischemia/reperfusion	[161]
NAD+	Bama miniature pigs (a swine model of ischemia/reperfusion injury)	20 mg/kg NAD+ or saline, i.v.	Before reperfusion	Dysinflammation, less cardiac fibrosis, and better ventricular compliance; reduced myocardial necrosis, and promoted cardiac function recovery	[162]
NAD+	Specific-pathogen-free male Sprague-Dawley rats	10 mg/kg i.p.	14 days	Attenuation the depression of cardiac function in the isolated rat hearts after ischemia-reperfusion	[163]
NMN	C57BL/6 wild-type mice	administration of 0.5 g/kg i.p.	30 min before ischemia and repetitive administration just before and during reperfusion	Reduced the infarct size after ischemia/reperfusion	[164]
NR	Male Wistar rats	i.v. infusion of 50 mg/kg NR	NR infusion for 5 min before and 15 min after the beginning of reperfusion	Restoration of small intestine microcirculation after mesenteric ischaemia/reperfusion; improved small intestine mucosa damage.	[167]
**Cardiomyopathy**					
NR soft pellets	*Srfh^−/−^* and Sf/Sf control littermates	Chow diet or NR-supplemented with 400 mg/Kg of body weight/day	50 days	Protection against cardiac dysfunction (measure LVEF and FS, dilatation and thinning of the LV wall)	[171]
NR soft pellets or in water	*Lmna^H222P/H222P^* and wild-type mice	Chow diet or NR-supplemented with 400 mg/kg of body weight/day	16 days	Increased NAD+ content in liver and heart and partially restore the left ventricular function and increase survival	[177]
NR soft pellets or in water	*Lmna*^H222P/H222P^ mice and wild-type mice	For post-symptomatic treatment, Lmna^H222P/H222P^ mice received 400 mg/kg per day orally by gavage	9 weeks	Stable left ventricular dimensions and fractional shortening	[177]
NAM	*Lmna*^H222P/H222P^ and wild-type mice	Received 500 mg/kg i.p.	every other day during 9 weeks	Treatment with NAM is not efficient to restore the cardiac NAD+ content and cardiac function	[177]
NR	C57BL/6 CD or HFD+L-NAME (HFpEF mice model)	400 mg/kg body weight/d	5 day per week for 4 weeks	Improved mitochondrial function, ameliorates cardiac hypertrophy, attenuates diastolic dysfunction, improves exercise capacity, and reduces lung capacity	[179]
NR medium and high dose	MutUNG1 mice and control littermates	CD, NR-supplemented with 400 mg/kg (medium dose) or 1,000 mg/kg (high dose)	2 weeks	NR high dose: inhibits SIRT3 activity due to an enhance levels of NAM and promotes mitochondrial dysfunction. Reduction of NAD+ levels in cardiac tissue and loss of mitochondrial deacetylation	[182]
NMN	Myh6-Cre:Klf4 fl/fl mice, designated CM-K4KO (C57BL/6J background)	500 mg/kg/day i.p.	5 days	NMN protected the mutant mice from pressure overload-induced HF	[194]
NR	Mouse model of cardiac hypertrophy established using Transverse aortic constriction surgery (C57BL/6J background)	400 mg/kg/day (daily oral gavage)	8 weeks	NR alleviated from cardiac hypertrophy and dysfunction	[202]
**Aortic Aneurysm**					
NR	Marfan Syndrome mouse model Fbn1c1039g/+	i.p. injections 1,000 mg/kg every second day	28 days	Elevation of TFAM levels, improvement of mitochondrial metabolism, and normalization of aortic function and diameter	[221]
NA NAM	*Apoe^−/−^* mice infused with Ang II and C57BL/6J mouse CaCl_2_ induced AAA	Drinking water supplemented with NA 0.3% *w/v*, NAM 0.1% or 0.4% *w/v*	2 days prior AAA induction to the end of the study	Protection against AAA formation mediated through NAD+ repletion that lead to SIRT1 activation	[24]
Me-NAM	*Apoe^−/−^* mice	Dietary supplementation of me-NAM (0.0057 and 0.017% *w/w*) in a high-fat diet containing 21% of fat and 0.2% cholesterol	8 weeks	Reduced aortic root atherosclerotic area	[30]
**Hypertension**					
NA	Male Sprague-Dawley rats	NA dissolved in the drinking water (50 mg/kg/day)	12 weeks	Ameliorated hypertension and partial reversal of upregulation of oxidative, inflammatory, and profibrotic mediators in the remnant kidney	[249]
NAD+	*Sirt1* transgenic and C57BL/6J mice	Incubation of NAD+ in whole aorta homogenates to measure SIRT1 deacetylase activity	80 min	Improvement of vascular remodeling in the aortas of transgenic mice resulting in a decreased SBP	[244]
NADH	Spontaneously hypertensive male rats	5 mg administered in single tablets daily	60 days	Lower SPB in treated rats vs. non-treated ones	[248]
β-NAD+ and NAM	Sea urchin egg	Incubation in the medium, Various concentrations of β-NAD+ (0–4 mM) and nicotinamide (0–5 mM)	90 min	vasorelaxation by inhibition of ADP ribosyl cyclase	[245]
β-NAD+ β-NADH NGD+ NGD and NAM	Male New Zeland white rabbit	Incubation in smooth muscle homogenates of pulmonary arteries β-NAD+ (2.5 mM) β-NADH (25 mM) NGD+ (250 µM) NGD+ (250 µM) + NAM (10 mM)	60 min	Vasorelaxation by inhibition of ADP ribosyl cyclase	[247]
NAM	Sprague-Dawley rats, wild-type C57BL/6 mice, and CD38/mice on a C57BL6 background	Injection of 6 mg/kg/min in the renal artery	3 min before and 5 min following endothelin-1 or sarafotoxin-6c injection	Vasorelaxation by inhibition of ADP ribosyl cyclase	[246]
NAM	Dahl salt-sensitive rats	40 mM oral supplementation in the drinking water	5 weeks	improved diastolic dysfunction	[31]
NAM	RUPP mice	Daily oral gavage (500 mg/kg/day)	In between 14.5 and 18.5 days postcoitus	Improving and preventing hypertension, fetal growth restriction, and premature birth	[250]
NAM	1-Non-pregnant female C57BL/6J 2-Pregnant female ICR 3-Asb4^−/−^ females	Daily oral gavage (500 mg/kg/day)	In between 12.5 and 18.5 days postcoitus	Improving and preventing hypertension, proteinuria, miscarriage, and premature birth in preeclampsia	[251]
NAM	1-C57BL/6J male mice 2-Renin-transgenic male mice 3-eNOS-null female and male mice	NAM dissolved in the drinking water (500 mg/kg/day) L-NAME: 50 mg/kg/day in drinking water L-NAME + NAM same dose as above in drinking water	60 days	Normalized blood pressure in mice with impaired eNOS function via suppressing inflammation	[252]
NMN	C57BL/6J mice	i.p. injection of NMN (500 mg/kg)	twice a day during 7 consecutive days	Preservation of myocardial NAD+ levels and functional compensation against pressure overload	[253]

## 5. NAD+-Increasing Strategies in Human Heart Health: From Bench to Bed

Clinical interest in NAD+-increasing strategies is growing. The use of these compounds offers exciting new biological insights and therapeutic opportunities. However, the results from clinical trials investigating the impact of vitamin B3 intermediate-based therapies on combatting cardiovascular diseases are still scarce (see ongoing clinical trials in Table 2). Thus, we next discuss current research focusing on the impact of NAD+ precursors on the cardiovascular system.

### 5.1. NA

Historically, NA was one of the first drugs used to treat atherogenic hyperlipidemia by virtue of its contribution to reducing LDL cholesterol levels and increasing HDL-C levels [254] (Section 1). The AIM-HIGH trial did not demonstrate a potential benefit of using NA to reduce cardiovascular events in patients with atherosclerotic cardiovascular disease and optimally controlled LDL cholesterol levels by statin therapy [255]. The potential of NA as an NAD+ source has been elusive in former clinical trials [19], and its clinical use has been progressively reduced mainly due to its adverse side effects, such as flushing [20], despite the generation of new formulations, i.e., a currently used PGD2 receptor subtype 1 (DP1) blocker developed with the purpose of decreasing NA side effects [256].

Pellagra is a systemic disease resulting from severe vitamin B3 deficiency. Although symptomatic cardiac involvement is rather infrequent in subjects with pellagra, a recent case of pellagra in a female child was reported to present signs of dilated cardiomyopathy, as revealed by echocardiography. Notably, NA replacement positively influenced cardiac dysfunction [257].

### 5.2. NAM

The clinical use of NAM may also have benefits in the treatment of clinical myocarditis. This condition refers to heart inflammation that can be caused by infectious (viral)-related mechanisms [258] and displays signs similar to those of other causes of inflamed hearts, including cardiac amyloidosis and hypertrophic cardiomyopathy [259,260]. Previous data in the middle of the 20th century demonstrated that NA was effective in the treatment of myocarditis diagnosed on the basis of abnormal electrocardiograms [261,262].

The latter data might be of particular clinical interest in the context of the COVID-19 pandemic. Indeed, myocarditis is one of the clinical findings observed in subjects with SARS-CoV-2 infection [263], and a positive effect of NAM or any other NAD+ precursor could be hypothesized to prevent this adverse cardiac outcome of COVID-19. Notably, SARS-CoV-2 has been recently reported to directly infect cardiomyocytes of patients with COVID-19 myocarditis but not cardiac macrophages, fibroblasts, or endothelial cells [264]. Similar results have also been found in vitro using iPSC-human derived cardiomyocytes [265]. Interestingly, NAD+ depletion may occur secondarily to the activities induced by NAD+-consuming enzymes, i.e., SIRT1 and PARP, which are directly involved in the activation of intracellular immune defenses upon SARS-CoV-2 infection. These data might suggest that NAD+ depletion is critical in the pathogenesis of viral myocarditis and that administration of NAD+ precursors may serve as a promising therapy. Consistently, vitamin B3 intermediates may be effective in promoting innate and adaptive responses to SARS-CoV-2 infection [266,267,268]. In particular, NAM treatment has been effective for reducing adverse disease outcomes of COVID-19 syndrome in other systems apart from the respiratory system in infected subjects, including the renal system [269]. Therefore, NAM and other NAD+ precursors, i.e., NR, can be hypothesized to contribute to NAD+ replenishment and attenuate myocardial damage. In this context, a recent clinical trial aimed to assess whether NR administration would affect the clinical course of the disease; however, myocardial structure or function was not considered an outcome measure (https://clinicaltrials.gov/ct2/show/NCT04407390, accessed on 20 October 2021).

NAD+ elevation using NAM is currently under investigation to assess its influence on myocardial injury in patients undergoing on-pump cardiac surgery (https://clinicaltrials.gov/ct2/show/NCT04750616, accessed on 20 October 2021) (Table 2).

### 5.3. NR

Because NR may be a more suitable NAD+ precursor than NA and NAM [270], the first clinical trials used NR to assess its pharmacokinetics in humans. Clinical trials involving NR supplementation have shown that orally administered NR is well tolerated with no adverse events [54,271]. Single doses of 100, 300 and 1000 mg of NR produced dose-dependent increases in the blood NAD+ metabolome [54].

Diabetic cardiomyopathy, a risk factor for HF, may occur even in well-controlled asymptomatic diabetic patients [272]. Because exogenous administration of NAD+ improves the mitochondrial NAD+ pool and cardiac function [175], its supplementation either directly or indirectly using NAD+ precursors has been proposed to contribute to increasing the mitochondrial NAD+ pool and mitochondrial homeostasis and in turn benefit myocardial health. However, the potential use of such therapies in obese and diabetic hearts requires further investigation [272].

Oral NR administration attenuated proinflammatory activation of PBMCs in 4 of 19 patients with HF [105]. This observation is consistent with findings following 6-week oral NR administration (1000 mg per day) in healthy middle-aged and older humans, which revealed that such treatment reduced SBP and aortic stiffness, two major independent risk factors for incident cardiovascular events and disease with advancing age [270]. However, these observations have not yet been reproduced in other studies and are being addressed in a currently ongoing clinical trial (https://clinicaltrials.gov/ct2/show/NCT03821623, accessed on 20 October 2021). As mentioned previously, in a long-term human cohort study involving patients with HF with HFpEF, high dietary intake of naturally occurring NAD+ precursors was associated with lower blood pressure and a reduced risk of cardiac mortality, suggesting a potential role of NAD+ precursors in treating diastolic dysfunction and HFpEF in humans [31].

Other therapeutic approaches have already been approved; for instance, a phase II clinical trial to assess the efficiency of NR for HF treatment is expected to start soon (https://anr.fr/Project-ANR-17-CE17-0015, accessed on 20 October 2021). Interestingly, an interventional clinical trial with 30 participants with systolic HF is underway to determine the safety and tolerability of NR (https://clinicaltrials.gov/ct2/show/NCT03423342, accessed on 20 October 2021) (Table 2), although no results have been published yet. Another interventional randomized placebo-controlled trial with NR treatment in elective left ventricular assist device surgery has been scheduled to demonstrate whether subjects randomized to receive NR will have higher myocardial NAD+ levels, improved mitochondrial function, restored gene expression, and reduced inflammatory responses compared to participants randomized to the placebo group (https://clinicaltrials.gov/ct2/show/NCT04528004, accessed on 20 October 2021) (Table 2).

### 5.4. NMN

A recent study performed in 48 young middle-aged recreationally trained runners supplemented with NMN for 6 weeks revealed that exercise training combined with NMN further elevated the ventilatory threshold in amateur runners, most likely due to enhanced O_2_ utilization of skeletal muscle rather than cardiac muscle [273]. Although cardiac function was not analyzed in this study, recent data in aged Drosophila demonstrated that exercise training can improve lipotoxic cardiomyopathy induced by a high-fat diet [274,275]. Because activation of the cardiac NMNAT/NAD+/SIR2/FOXO and NMNAT/NAD+/SIR2/PGC-1α pathways was identified to mediate the beneficial effects of endurance training in this animal model, NMN administration can be hypothesized to further potentiate exercise resistance to high-fat diet-induced cardiomyopathy. Further investigation is warranted to directly address the potential positive influence of NMN on heart health in future clinical trials.

**Table 2:** Current clinical trials of NAD+-increasing strategies to combat cardiovascular diseases.

Interventions	Aimed Cardiovascular-Related Outcomes	Study Start Date	Finish Date	Time Frame	Dosage	NCT
NA	Change in the mean severity of proximal stenosis	Jan 1984	Aug 1989	2.5 years	NA was started at 125 mg twice a day and gradually increased to 500 mg four times a day (with meals and at bedtime) at one month and 1 g four times a day at two months. If the LDL cholesterol level did not fall below 3.1 mmol per liter (120 mg per deciliter) after three months, the dose of niacin was increased to 1.5 g (three tablets) four times a day, but no further	NCT00000512
NA	Change in minimal diameter of coronary artery lesions	Dec 1986	Nov 1992	Not available	Not available	NCT00000461
NA	Change in proximal obstructive disease	Sep 1994	Aug 1999	2.5 years	Not available	NCT00000553
NA	Change in plaque morphology	Jan 2000	Sep 2005	12 months	NA 20 mg daily	NCT00307307
NA	Changes in carotid plaque composition	Jun 2001	Apr 2019	40 months	NA 2000 mg daily	NCT00715273
NA	Inflammation and clot formation and blood vessel health	Jun 2002	Nov 2005	16 weeks	NA 1500 mg daily	NCT00590629
NA	Endothelial function	Jun 2003	Jun 2005	16 weeks	NA 1.5 g daily	NCT01921010
NA	Changes in aortic and carotid plaque architecture and composition	Sep 2003	Dec 2008	18 months	NA extended release 0.5 to 3.0 g daily	NCT00127218
NA	Change in superficial femoral artery wall volume	Apr 2004	Dec 2021	24 months	NA 1500 mg daily	NCT00687076
NA	Brachial artery flow mediated dilation	Sep 2005	Aug 2008	3 months	NA extended release 1500 mg daily	NCT00150722
NA	Relative effect on flow-mediated dilatation of radial artery	Mar 2006	Jun 2009	6 months	NA extended-release	NCT00298909
NA	Endothelial function by high resolution echography in response to nitric agent	Jun 2007	May 2009	3 months	Not available	NCT00855257
NA	Mean plaque lipid composition in carotid arteries	Mar 2008	Feb 2015	24 months	NA extended-release 1500 mg or 2000 mg daily	NCT01178320
NA	Composite score of plaque inflammation/stability, plaque instability protein composite score, total cholesterol, and free cholesterol	Apr 2009	Oct 2010	12 weeks	NA extended-release tablet 2 g daily	NCT00804843
NA	Endothelial dependant dilatation of the arterial wall	Sep 2010	Oct 2011	12 weeks	NA 2000 mg/40 mg	NCT01126073
NA	Change in percent atheroma volume by intravascular ultrasonography	Oct 2010	Nov 2015	12 months	NA extended release 1500–2000 mg daily	NCT01221402
NA	Change from baseline in arterial fluorodeoxyglucose uptake	Mar 2012	Jan 2013	12 weeks	NA titrated to 6000 mg daily	NCT02003638
NA	Changes in protein or lipid composition of any lipoprotein fraction and changes in vascular compliance	Mar 2015	Jun 2019	14 weeks	NA extended release 2000 mg/day	NCT02322203
NAM	Number of participants with adverse events (early onset Preeclampsia)	Aug 2014	Nov 2018	48 h	Either 500 mg or 1000 mg by mouth each morning until delivery or 14 days, whichever occurs first	NCT02213094
NAM	NAD+ augmentation in cardiac surgery associated myocardial injury trial. Troponin T (area under the curve)	Feb 2021	Sep 2021	From baseline to three days after surgery	3 g on the day of surgery and post-surgical days one and two	NCT04750616
NR	Mean IL-1beta release From peripheral blood mononuclear cells during refeeding after 24 h fast	Jun 2016	Aug 2018	4 weeks	Either NR at 1000 mg/day or placebo for one week, followed by a washout period of 2–3 weeks, then a crossover to placebo or NR at 1000 mg/day for one additional week. The end point was analyzed at end of each treatment.	NCT02812238
NR	Bioavailability (pharmacokinetics), safety (blood pressure, pulse, etc.) and impact on mitochondrial disease symptoms *	Dec 2017	Dec 2019	24 h (bioavailability and safety) and 4 weeks (mitochondrial characteristics)	not defined (open-label experimental medicine study; all subjects will receive the same dosage of the supplement)	NCT03432871
NR	Number of participants without heart failure linked inflammation in patients with stable, systolic heart failure	Jun 2018	Oct 2020	12 weeks	Starting at 500 mg daily (250 mg BID) be increased at two weekly intervals by 250 mg/dose (BID) (500 mg/day) to a final dose of 1000 mg PO BID (2000 mg/day)	NCT03565328
NR	Incidence of treatment-emergent adverse events (safety and tolerability), whereby the main aim was to assess the preoperative effect of NR supplementation in patients undergoing elective left ventricular assist device (LVAD) implantation	Nov 2018	Sep 2020	Up to 14 days	Dose Escalation: Day 1: 250 mg (1 capsule) twice daily (total daily intake = 500 mg) Day 2: 500 mg (2 capsules) twice daily Day 3: 1000 mg (4 capsules) twice daily (total daily intake = 2000 mg) Dose Maintenance: Day 4: 1000 mg (4 capsules) twice daily Day 5–14 as applicable through Day Before Surgery: 1000 mg (4 capsules) twice daily(total daily intake = 1000 mg)	NCT03727646
NR	Exploratory endpoint: effect of NR on left ventricular diastolic and systolic function	May 2019	Jun 2019	12 weeks	The initial dose will be 1 capsule twice daily, followed by weekly up-titration by 1 capsule/dose to a final dose of 4 capsules (1000 mg) twice daily at the end of week 4; participants will be continued on the final dose up to the final follow up visit (week 12)	NCT03423342
NR	Carotid-femoral pulse wave velocity (primary) and systolic and diastolic blood pressure (secondary)	Nov 2019	Sep 2014	3 months	500 mg by mouth twice	NCT04040959
NR	Systolic blood pressure	May 2019	Dec 2023	3 months	500 mg of the vitamin B3-precursor, nicotinamide riboside (NIAGEN) twice per day (1000 mg per day total)	NCT03821623
NR	Between-group comparisons of myocardial NAD(H) levels, myocardial mitochondrial morphology, myocardial mitochondrial respiratory function, myocardial protein acetylation, myocardial gene expression by RNA-seq and the myocardial epigenome by ATAC-seq, inflammatory markers in myocardium	Sep 2020	Aug 2024	Up to 14 days	Dose Escalation: Day 1: 250 mg (1 capsule) twice daily (total daily intake = 500 mg) Day 2: 500 mg (2 capsules) twice daily Day 3: 1000 mg (4 capsules) twice daily (total daily intake = 2000 mg) Dose Maintenance: Day 4: 1000 mg (4 capsules) twice daily Day 5–14 as applicable through Day Before Surgery: 1000 mg (4 capsules) twice daily(total daily intake = 1000 mg)	NCT04528004
NR	Change in Systolic blood pressure (primary), and change in arterial stiffness (secondary)	Jul 2020	May 2022	6 week	1000 mg/day	NCT04112043
NMN	Effect of NMN on flow mediated dilation and brachial-ankle pulse wave velocity	Jun 2021	Jul 2022	2 months	NMN10,000 WRIGHT LIFE^®^ + lifestyle modification	NCT04903210
NR	Vasodilatory Reserve (Percent change in systemic vascular resistance at baseline vs. exhaustion) and Kansas City Cardiomyopathy Questionnaire Overall Summary Score (Assess the impact of our interventions on quality of life)	Oct 2021	Sep 2026	6 days	Potassium Nitrate (KNO3) 6 mmol three times daily + Propionyl-L-Carnitine (PLC) 1000 mg twice daily + NR 300 mg three times daily	NCT04913805

* studies were focused in mitochondrial diseases, including myopathies, in skeletal, not cardiac, muscle. Abbreviations were used: NAD: nicotinamide adenine dinucleotide; NA: nicotinic acid; NAM: nicotinamide; NMN: nicotinamide mononucleotide; NR: nicotinamide riboside; NCT, clinical trial identifier (ClinicalTrials.gov).

## 6. Concluding Remarks

In addition to the long time since the discovery of NAD+, a huge number of studies have been performed, although more work is required to fully understand its role in human diseases. Compelling evidence suggests that NAD+ homeostasis is required for normal cardiovascular function. Numerous studies suggest that declining NAD+ concomitant with mitochondrial dysfunction is a common trait in myocardial and cardiovascular diseases. Over the last few decades, the contribution of NAD+ precursor supplementation to increasing NAD+ levels and improving cardiovascular function in multiple preclinical animal models of several cardiovascular pathologies has attracted therapeutic interest [276]. Furthermore, except for NA, which causes some adverse clinical outcomes, vitamin B3 intermediates have been demonstrated to be safe and to have promising beneficial effects in combating cardiovascular diseases in vivo. Additional basic and translational research is needed to better understand the underlying mechanisms and consequences for cardiac and vascular structure and function. From a translational perspective and despite considerable current efforts, many questions remain in relation to the use of NAD+ precursors, including administration, optimal and safe doses, and the distribution of NAD+ in different tissues, to achieve the desired therapeutic efficacy. Recent and ongoing clinical trials are predominantly investigating NR, followed by NAM and then NMN, while NA is studied the least. Whether these NAD+-increasing therapies will favorably influence cardiovascular outcomes remains to be determined.
